# Correlates of mobile screen media use among children aged 0–8: protocol for a systematic review

**DOI:** 10.1186/s13643-016-0272-y

**Published:** 2016-06-03

**Authors:** Susan Paudel, Justine Leavy, Jonine Jancey

**Affiliations:** Maharajgunj Medical Campus, Institute of Medicine, Maharajgunj, Kathmandu Nepal; Collaboration for Evidence, Research and Impact in Public Health (CERIPH), School of Public Health, Curtin University, Perth, Western Australia Australia

**Keywords:** Mobile screens, Children, Interactive media, Systematic review, Protocol

## Abstract

**Background:**

Childhood is a crucial period for shaping healthy behaviours; however, it currently appears to be dominated by screen time. A large proportion of young children do not adhere to the screen time recommendations, with the use of mobile screen devices becoming more common than fixed screens. Existing systematic reviews on correlates of screen time have focused largely on the traditional fixed screen devices such as television. Reviews specially focused on mobile screen media are almost non-existent. This paper describes the protocol for conducting a systematic review of papers published between 2009 and 2015 to identify the correlates of mobile screen media use among children aged 0–8 years.

**Methods:**

A systematic literature search of electronic databases will be carried out using different combinations of keywords for papers published in English between January 2009 and December 2015. Additionally, a manual search of reference lists and citations will also be conducted. Papers that have examined correlates of screen time among children aged 0–8 will be included in the review. Studies must include at least one type of mobile screen media (mobile phones, electronic tablets or handheld computers) to be eligible for inclusion. This study will identify correlates of mobile screen-viewing among children in five categories: (i) child biological and demographic correlates, (ii) behavioural correlates, (iii) family biological and demographic correlates, (iv) family structure-related correlates and (v) socio-cultural and environmental correlates. PRISMA statement will be used for ensuring transparency and scientific reporting of the results.

**Discussion:**

This study will identify the correlates associated with increased mobile screen media use among young children through the systematic review of published peer-reviewed papers. This will contribute to addressing the knowledge gap in this area. The results will provide an evidence base to better understand correlates of mobile screen media use and potentially inform the development of recommendations to reduce screen time among those aged 0–8 years.

**Systematic review registration:**

PROSPERO CRD42015028028.

**Electronic supplementary material:**

The online version of this article (doi:10.1186/s13643-016-0272-y) contains supplementary material, which is available to authorized users.

## Background

Use of electronic media, including television, mobile phones and tablets, is a major part of children’s lives [1]. Young children are surrounded by televisions, computers and newer technologies such as smartphones and tablets [2]. Recently, there has been a significant increase in the use of mobile screen media (smartphones and tablets) among young children [3, 4]. Touch-screen devices easily attract children, and even from a short exposure, children become expert users [5]. Furthermore, parents are increasingly using these devices to pacify their children, with nearly one in ten parents giving smartphones or tablets to their children when they are undertaking household chores [4]. Findahl [6] reports that 50 % of 3- to 4-year-old Swedish children use tablets whereas one in four children uses smartphones. A US-based study found around one third of toddlers were using mobile phones for 30 min every day [7]. Likewise, 16 % of Australian children aged 2 to 4 have at least one screen-based electronic device in their bedroom [8].

Research indicates that limiting exposure to electronic media of all forms reportedly lowers attention problems, improves diet and sleep quality, reduces risk of obesity [9–12] and increases levels of physical activity [13, 14]. Conversely, increased duration of screen time among young children is positively associated with delayed fundamental motor skill (FMS) development [15]. Yet, mastery of FMS during childhood is crucial for life-long engagement in physical activity [16, 17]. Not surprisingly, recent Australian studies have reported poor locomotor and object control skills among boys and girls [18], which may be influenced by increasingly high screen time and low engagement in physical activity.

Both the Australian Government’s Department of Health and the American Academy of Paediatrics recommend that children under 2 years not spend any time on any form of screen-viewing, such as smartphones and tablets, computers, television or other electronic media [1, 19]. For Australian children 2 to 5 years, the screen time should be less than 1 h per day [19]. However, evidence indicates that young children of this age already have high levels of screen time with the majority exceeding these recommendations [20]. Nearly three quarters of Australian children aged 2 to 4 exceed the screen time recommendation of ≤1 h per day [8]. On average, they spend 112.5 min on screen-based entertainment (television, electronic games or the Internet) every day, with no significant difference between boys and girls [21]. This has potential to impact on longer term behaviour with this greater duration of screen time in early childhood being associated with greater screen time at school age [9]. Furthermore, such habits have a strong tracking to adulthood [10].

Mobile device use by children is an important issue because of the portability and increasing presence resulting in greater user engagement and presence of media in all aspects of children’s lives [22]. Despite the widespread adoption and use of these mobile screen devices by children, very little research regarding their use and impact has been undertaken [22]. Considering the developmental differences and the influence of family and environmental factors on shaping the behaviours of these young children, identification of correlates specific to this group is vital. However, most of the research and recent reviews focus on traditional media, particularly television. These reviews report that correlates of children’s screen time are multi-dimensional [23]. Some of these correlates include maternal depression, parental support and stimulation, child body mass index (BMI), parental television viewing, media access, limits on media use at home and socio-economic status [24, 25]. Systematic reviews which have studied the correlates associated with mobile screen media use among young children are almost non-existent.

Duch et al. [24] and Vanderloo [26] have used the bio-ecological model to review the correlates of screen time involving traditional screen media such as televisions and computers. This model provides a strong theoretical basis to understand health behaviour change, facilitating better understanding of factors associated with screen use in these reviews. The model states that human development is affected by intrapersonal factors (e.g. human biology and genetics) to interpersonal factors (e.g. families and peers) and distant factors (e.g. culture, community and environment) [27, 28]. It assumes that there is significant interaction between different layers of influence in shaping human behaviour [28]. This review will also be informed by the bio-ecological model which will add to the small body of literature that already exists and will contribute to understanding the difference in factors determining traditional media versus mobile media use. As suggested by the model and reflected in previous reviews, this study will identify correlates of mobile screen-viewing among children in five categories: (i) child biological and demographic correlates, (ii) behavioural correlates, (iii) family biological and demographic correlates, (iv) family structure-related correlates and (v) socio-cultural and environmental correlates.

With the increasing ownership of these newer technologies, it is very likely that their use will continue to increase, particularly considering the ease of accessibility associated with their size, design and mobility. Therefore, identifying correlates of screen time specific to young children is crucial to understanding the issue and to informing interventions to potentially reduce screen time in this age group. This systematic review aims to identify correlates of screen time (specifically smartphones, electronic tablets, handheld computers, personal digital assistants (PDA) and any other form of mobile screen media) among young children (0–8 years) in papers published between January 2009 and December 2015.

## Methods

This systematic review will be guided by the Preferred Reporting Items for Systematic Reviews and Meta-Analyses (PRISMA) [29] statement to ensure transparency in the selection of papers and improved reporting [29–31]. This protocol has also been developed using the PRISMA Protocols 2015 (PRISMA-P-2015) [32] as explained in Additional file 1. This protocol will be strictly adhered to for the review, and any differences between the protocol and the review will be reported along with the rationale for the difference. In order to avoid unintended duplication, this study is registered with PROSPERO International Prospective Register of Ongoing Systematic Reviews (CRD42015028028).

### Outcome measure

Mobile media-related screen time is the outcome variable of interest, and this study aims to identify the correlates of mobile screen time among children less than 8 years. Screen time in the study will refer to the total amount of time spent in front of portable screens, for example mobile phones, electronic tablets, handheld computers, PDA and any other form of mobile electronic media. Correlates refer to the variables associated with increase or decrease in mobile screen media use. Considering the increasing use of mobile screens which have started replacing the traditional fixed screens such as televisions and computers, the focus of this study will only be on mobile devices.

### Information sources and search strategy

To identify published primary research articles on correlates of screen time among children, a literature search of electronic databases will be carried out. Electronic databases Medline, Scopus, Embase, CINAHL Plus, PubMed, ProQuest, PsycINFO and Web of Science will be searched. Database search will be limited to papers published from January 2009 to December 2015. Child-related keywords (child*, preschool, infant, kid and toddler) and screen-related keywords (screen time, screen viewing, mobile phone, cell phone, smartphone*, PDA, tablet*, iPad*, handheld media, handheld computer*) will be used to locate potential papers in these databases. Additionally, reference lists and citations will be manually searched. Grey literature will not be searched. An example of a search strategy using the CINAHL Plus database is presented in Additional file 2: Table S1 [2].

The search will be limited to papers published in English with no restriction on study design. Study participants will be limited to children or parent-child dyads or parental reports. Both published and in-press papers will be searched. To ensure that all relevant papers have been identified, Google Scholar profile of authors with frequent publications in this domain will be cross-checked.

### Types of studies

Considering the dearth of research in this area, studies of all designs, from randomised controlled trials to quasi-experimental trials, cohort studies, case-control studies and cross-sectional studies, will be included. However, qualitative studies, systematic reviews and meta-analyses will be excluded.

### Inclusion criteria

Quantitative primary research articles, published in the English language, from January 2009 to December 2015 that have explored correlates of screen time of mobile devices among children under 8 years of age will be included in this review. Studies carried out in home or community settings with children, parents or parent-child dyads as the study participants will be included. Childcare centre-based studies will be excluded. Studies carried out among unhealthy participants and children above 8 years will not be included. Likewise, studies that have investigated screen time but do not include any one form of mobile device will be excluded. Similarly, studies with an older age group and no sub-group analysis for the target population will also not be included. The research question is presented in a population, exposure, comparison and outcome (PECO) format in Table 1.

**Table 1:** Research question using PECO format

Criteria	Description
P: population	Children under 8 years
E: exposure	Correlates of mobile screen media use
C: comparison	With vs. without the correlates
O: outcome	Use of mobile screens (e.g. mobile phones, electronic tablets, handheld computers, personal digital assistants (PDA) and any other form of mobile electronic media)
Types of studies	All designs (cross-sectional, case-control, cohort and intervention studies), only quantitative studies
Exclusion	Papers that have not studied correlates of screen time of mobile devices
	Studies that have studied sedentary behaviour but not explicitly addressed screen time
	Studies that have not included at least one form of a mobile screen device
	Systematic reviews and meta-analysis
	Grey literature
	Qualitative studies
	Studies carried out in settings other than home or community
	Studies carried out among unhealthy participants
	Studies with larger age group with no sub-group analysis for the target group
	Papers published before 2009 and after December 2015
	Papers published in language other than English
	Non-peer-reviewed articles
	Studies involving children older than 8 years

### Screening

All the identified articles, through both database and manual searching, will be exported to Endnote x7 citation management software [33]. Duplicate articles will be identified using the option provided by the software and will be removed. The PRISMA flowchart will guide the screening and reporting of the process. At the first stage, all the non-relevant titles will be removed. The remaining articles will then be subjected to abstract screening. The abstracts will be screened against the inclusion criteria by two researchers, and all non-relevant abstracts will be removed. Full texts of the remaining articles will be retrieved. All the three researchers (SP, JJ and JL) will review these full texts against the inclusion/exclusion criteria to assess their eligibility for inclusion. Joint meetings will be held to review the individual results and establish consensus. In the event of any differences, the article will be re-read jointly and its eligibility for inclusion ascertained.

### Quality appraisal

A modified version of the checklist by Downs and Black [34] will be used to assess the quality of studies and risk of bias. Out of 27 items suggested in the checklist in the themes of reporting, external validity, internal validity-bias and internal validity-confounding (selection bias), relevant items will be used for this study. Ten items (questions 1–3, 6, 7, 10–12, 18, 20) are considered to be appropriate for this review and are consistent with previous reviews using this checklist [24]. A score of ‘1’ will be allocated for ‘yes’ and a score of ‘0’ will be allocated to ‘no’ and ‘unable to determine’. Out of 10 possible points, a total score of greater than 5 will indicate a good-quality paper. Quality appraisal will be independently carried out by at least two of the researchers, and in the event of any discrepancies in rating, the third researcher will be consulted to reach consensus.

### Data extraction and management

A data extraction table will be developed and used to maintain consistency and avoid bias in extraction. From all the studies deemed suitable for inclusion, information on study design, country of study, age group of children, sample size, type of screen media studied, method of assessing screen-viewing and the correlates studied will be extracted. Association between correlates and mobile screen use will be retrieved from the results of the study based on the adjusted measures of association (odds ratio or relative risk).

### Data synthesis, analysis and reporting

The exposure variables (correlates) will be grouped into five categories: (i) child biological and demographic correlates (sex, child’s age, ethnicity, birth order), (ii) behavioural correlates (duration of media exposure, onset age of media exposure), (iii) family biological and demographic correlates (parent’s age, education, occupation, economic status), (iv) family structure-related correlates (number of siblings) and (v) socio-cultural and environment correlates (variables like place of residence, parental media use, access to media at home) [24, 26]. This grouping is based on the bio-ecological model, separating correlates to proximal and distal factors. As suggested by the model, child biological, demographic and behavioural correlates will represent intrapersonal factors; family biological, demographic and structure-related correlates will represent interpersonal factors, whereas socio-cultural and environmental correlates are the distal factors [24, 26–28]. Mean age of the children and mean daily screen time will be calculated based on availability of such information in the selected papers. Statistical analysis, if any, will be carried out using STATA software (Version 13, Stata Corporation, College Station, TX, USA).

## Discussion

To date, most of the studies on children’s screen time have focused on the traditional screen media such as television and video. Hence, the television has dominated the majority of the screen time studies in the past decade. In view of the pervasive increase in access and use of the modern mobile devices, which have now become an important part of children’s lives, a focus on portable mobile electronic media is needed. This study will identify the correlates associated with mobile media use among young children up to 8 years through a systematic review of published peer-reviewed papers. This will contribute towards addressing the knowledge gap in this area and provide an evidence base to better inform the development of policy and practice to reduce screen time among children aged less than 8 years.

## Abbreviations

PDA, personal digital assistants; PRISMA, Preferred Reporting Items for Systematic Reviews and Meta-Analyses

## Additional files

Additional file 1: PRISMA-P (Preferred Reporting Items for Systematic review and Meta-Analysis Protocols) 2015 checklist: recommended items to address in a systematic review protocol. (DOC 83 kb) Additional file 2: Table S1. An example search strategy in CINAHL Plus database. (DOCX 13 kb)
