# Supplementary material for: Correlates of mobile screen media use among children aged 0–8: protocol for a systematic review
Source: Syst Rev. 2016 Jun 3;5:91. doi: 10.1186/s13643-016-0272-y (PMC4891911; doi:10.1186/s13643-016-0272-y)
Supplement: Additional file 2: Table S1. — An example search strategy in CINAHL Plus database. (DOCX 13 kb) [file 13643_2016_272_MOESM2_ESM.docx]

**Table S1: An example search strategy in CINAHL Plus database**

| Database name | **CINAHL Plus** | |
| --- | --- | --- |
| Search date | 20^th^ October 2015 | |
| Keyword (include related topics) | Child, preschool, toddler, kid, screen time, mobile, tablets, smartphones | |
| Actual Search strategy | TI ( Child* or preschool* or kid* or toddler* or infant* ) AND TI ( mobile phone* or cell phone* or smartphone* or tablet* or iPad* or smartboard* or handheld computer* or computer* or screen time or mobile screen or touchscreen* )  **Limiters**  - Published Date: 20000101-20151231; English Language; Peer Reviewed; Research Article; Human; Age Groups: All Child; Language: English  **Expanders**  - Search related subjects  **Search modes** - Boolean/Phrase | 255 |
|  | TI ( Child* or preschool* or kid* or toddler* or infant* ) AND TI ( mobile phone* or cell phone* or smartphone* or tablet* or iPad* or smartboard* or handheld computer* or computer* or screen time or mobile screen or touchscreen* )  **Limiters**  - Published Date: 20000101-20151231; English Language; Peer Reviewed; Research Article; Human; Age Groups: All Child; Language: English, **Exclude MEDLINE records**  **Expanders**  - Search related subjects  **Search modes** - Boolean/Phrase | **82** |
| Name of EndNote library | Literature search_Cinahl | |
| Number of records loaded into EndNote library | 82 | |
